# Effects of a supervised exercise program in addition to electrical stimulation or kinesio taping in low back pain: a randomized controlled trial

**DOI:** 10.1038/s41598-022-14154-5

**Published:** 2022-07-06

**Authors:** María Encarnación Aguilar-Ferrándiz, Guillermo A. Matarán-Peñarrocha, Rosa María Tapia-Haro, Yolanda Castellote-Caballero, Celia Martí-García, Adelaida María Castro-Sánchez

**Affiliations:** 1 Biosanitary Research Institute (Instituto de Investigación Biosanitaria) ibs. GRANADA. Department of Physical Therapy, Faculty of Health Science, University of Granada, Granada, Spain; 2 Family Medicine and Primary Care, Andalusian Health Service, Distrito Sanitario Málaga, Málaga, Spain; 3 Department of Physiotherapy, Faculty of Health Science, University of Jaén, Jaén, Spain; 4 Nursing Department, Faculty of Health Sciences, University of Málaga, C/Arquitecto Francisco Peñalosa, 3, 29071 Málaga, Spain; 5 Department of Nursing, Physical Therapy and Medicine, University of Almería, Almería, Spain

**Keywords:** Pain management, Rehabilitation

## Abstract

Chronic low back pain it is one of the most common health problems worldwide. Usually is accompanied by a complex set of symptoms and generates significant direct and indirect socioeconomic and health costs. From a therapeutic point of view, there are a wide variety of methods to address the treatment of this pathology, however, these therapies have not been shown definitive efficacy. To investigate the effect of a mixed treatment with exercise and electrical stimulation versus exercise and kinesio taping in patients with non-specific chronic low back pain. A total of 58 patients participated in this single-blinded randomised clinical trial. Participants were assigned to the exercises- kinesio taping group, or exercises- analgesic current group, both received 12 treatment sessions. Disability, fear of movement, anxiety, depression, sleeps quality, pain, lower limb mechanosensitivity and pressure-pain thresholds were recorded at baseline and after 4 weeks of treatment. The 2 × 2 mixed analysis of covariance test showed statistically significant differences between groups for pain (*P* = 0.046). Pair-wise comparisons with baseline demonstrated significant differences for both groups in pain (*P* ≤ 0.001), disability (*P* ≤ 0.001), pressure-pain thresholds (*P* ≤ 0.044), lower limb mechanosensitivity, (*P* ≤ 0.047), anxiety (*P* ≤ 0.001), depression (*P* ≤ 0.001) and sleep quality (*P* ≤ 0.010). Patients with chronic low back pain who received a combined treatment of exercises and kinesio taping or analgesic current showed an improvement in pain, disability, anxiety, depression and sleep pattern. Moreover, exercises combined with electrotherapy produces greater improvements over these variables.

Trial registration: NCT02812459.

## Introduction

Low back pain (LBP) has been one of the biggest public health problems, affecting up to 80% of the general population at least once in their life^1^. Scientific literature has demonstrated that LBP is accompanied by a complex set of symptoms such as physical functional disturbs, pain, fatigue, sleep deficits, poor quality of life, and altered emotional well-being^2^. The probability of being free of pain 12 months after having lumbar pain is only 42%^1^, so there is an urgent need for more effective treatments. In this case, the pain becomes chronic (CLBP) to the point of being considered the main cause of years lived with disability^3–5^. This pain is classified in most cases as “non-specific” pain, making it difficult to attribute it to a specific pathology^6^.

Several treatments for LBP have been studied, including education programs^7^, chiropractic therapy^8^, kinesiology^9^, exercise^10^, health counseling^11^, manipulative spinal therapy^12^, medication^13^ and electrotherapy^14^. Within the exercise program, lumbopelvic stabilization work is effective in chronic LBP, fundamentally from the point of view of the disability generated (work absenteeism)^15^. However, its effects are more limited in terms of subjective perception of pain. Therefore, together with the completion of these exercises, another series of complementary analgesic measures will be required^15^.

Kinesio taping (KT) has been previously used as a complementary technique for the clinical management of patients with chronic pain^16,17^, as it provides a beneficial effect by relieving pain while not constricting movement^18^. Four beneficial effects of KT have been defined: normalization of muscle function, increase of lymphatic and vascular flow, reduction of pain and contribution to possible corrections of misalignments^17^. Although the degree to which these mechanisms contribute to some clinical effects is unknown, some studies have shown a significant beneficial effect, compared to placebo applications^1^. The results of its use could be contradictory when it refers to some parts of the body, but in general, the literature shows consistency regarding the benefits of the use of KT for the relief of CLBP^19^.

Another technique used would be electrotherapy^14,20,21^. Electroanalgesia with transcutaneous electrical nerve stimulation (TENS) is one of the most used for the relief of chronic and acute pain^20,21^. TENS consists in the application of low frequency electrical impulse currents through the skin to stimulates the peripheral nerves and produce various physiological effects^21^ which includes the affectation of the descending pathways in the cerebral cortex promoting the release of substances such as enkephalin and endorphins^22^ and the potential of being able to inhibit nociceptive fibbers, according to the gate control theory of pain^23^.

To the best of our knowledge, there is no further previous evidence from studies based on the comparison of these treatment techniques (i.e. KT and TENS) for the control of pain and disability perception in patients with non-specific CLBP.

Considering the mechanisms of action of the previously mentioned techniques and the existing evidence on the results for the relief of non-specific CLBP, we hypothesize that a combined intervention of TENS and exercise will be more effective in reducing pain and, therefore, will improve other factors such as sleep quality, than the combined application of KT and exercises.

## Methods

### Aim

Considering this background, the purpose of this this study was to analyse the effectiveness of KT and electrical stimulation in combination with exercise in people with non-specific CLBP.

### Design

A single-blind randomized clinical trial with parallel design (allocation ratio 1:1) was conducted in patients with non-specific CLBP, who were referred to the clinical laboratory of the Physiotherapy Department of the University of Granada from a private Physiotherapy Centre in Granada, and who were studied between June 2016 and April 2018.

### Participants

Study inclusion eligibility criteria were: age between 25 and 65 years, presence of CLBP (defined as pain and discomfort located below the rib flange, persistent for 12 weeks or more^24^) for 3 months or more, not currently receiving physical therapy and a score of four points or more on the Roland Morris Disability Questionnaire (RMDQ)^25^. Exclusion criteria were: the presence of lumbar stenosis; any clinical signs of radiculopathy; a diagnosis of spondylolisthesis and/or fibromyalgia; treatment with corticosteroid or oral medication within the past 2 weeks; a history of spinal surgery; disease of the central or peripheral nervous system.

Informed consent was obtained from all the participants before their involvement. The study was approved by the bioethics committee of the University of Granada (Spain) on 2016 and complied with the 2013 modification of the Helsinki Declaration and Spanish legislation for clinical trials (ClinicalTrials.gov Identifier: NCT02812459).

### Self-reported outcomes

Subjects provided demographic, clinical information and completed self-report measures including: the Spanish version of the RMDQ^26^ for assessing disability due to CLBP, the Oswestry Disability Index (ODI)^27^ to check the level of functional impediment patients experienced as a result of back pain, Tampa Scale for Kinesiophobia (TSK)^28^ for assessing fear of movement, the Beck Depression^29^ and Anxiety^30^ Inventories, the Pittsburgh Sleep Quality Index (PSQI)^31^, and Numerical Pain Rating Scale (NPRS)^32^ for pain.

The primary outcome of this study was the change from baseline to post-treatment at 1 month in the RMDQ. The RMDQ is a widely used well-validated measure with good reliability for assessing disability due to LBP^25,26^. It is a self-administered measurement scored on a 24-point scale from 0 = no disability to 24 = severe disability where a 2–3 point change from baseline is considered a minimum clinically important difference (MCID)^25^. We used the Spanish version of the questionnaire, which has been shown a good test–retest reliability (ICC: 0.87) and internal consistency (Cronbach α: 0.84–0.91)^26^.

#### The secondary outcome measures were

The ODI^27^, that evaluates daily life activity limitations in 10 dimensions, each scored on a 6-point scale (0–5 points); the total points scored are expressed as a percentage, used to classify individuals as minimally disabled (0–10%), moderately disabled (20–40%), severely disabled (40–60%), crippled (60–80%), or bedbound (80–100%)^27^. The Spanish version of the ODI has shown good test–retest reliability (ICC: 0.92) and favorable internal consistency (Cronbach α: 0.86)^27^. Ostelo et al.^33^ reported that a change of 10 points is considered as the MCID for the ODI.

Change from baseline in TSK^28^, comprising 17 items on the fear of movement or recurrent lesion, each scored on a 1–4-point Likert scale from “completely disagree” to “completely agree” where higher values reflect greater fear of (re)injury^28^. Test–retest reliability ranged from 0.90 to 0.96 in patients with chronic LBP^28^. The Spanish version has a good reliability (internal consistency and stability) and validity (convergent and predictive) ^28^.

Beck’s Anxiety Inventory^30^ consists of 21 items that are scored on a scale that goes from “not at all” to “severely”. The total score is interpreted according to the following classification: 0–7 indicates minimum anxiety, 8–15 mild anxiety, 16–25 moderate anxiety, and 26–63 severe anxiety. Beck’s depression inventory^29^ is made up of 21 items to evaluate the intensity of depression. In each of the items the subject has to choose a sentence from a set of four possible answers (punctuated with 0–1–2–3). The total score of the 21 items varies from 0 to 63. A persistent score of 17 or more indicates the possible need for professional help.

The PSQI^31^ contains a total of 19 questions, grouped into 10 sub-questions. The 19 questions combine to form seven areas with their corresponding score, each of them which shows a range between 0 and 3 points. In all cases, a score of “0” indicates ease, while a score of 3 indicates severe difficulty, within their respective area. The score of the seven areas is finally added to give a global score ranging from 0 to 21 points.

Change from baseline in the NPRS^24^, which is a Visual Analogue Scale for pain intensity ranged from 0 = no pain to 10 = worst imaginable pain. It was used to assess the patients’ current level of pain and the worst and lowest level of pain experienced in the preceding 24 h^32^. The MCID for the NPRS in patients with CLBP has been reported to be 2.5points^33^.

### Physical outcomes

To test the lower limb mechanosensitivity we used the Straight Leg Raise^34^ (SLR) and Slump Tests (ST)^35^. For the SLR, the degree of hip flexion was measured with a large universal goniometer placed lateral to the pelvis, with the proximal arm parallel to the patient’s trunk and the distal arm lateral to the thigh, in line with the femoral condyle^34^.

For the ST^35^, the degree of knee extension was measured with an universal goniometer placed with the fulcrum located on the knee, with the fixed arm (proximal) aligned with the trochanter, and the mobile arm aligned with the lateral peroneal malleolus. The ST is only considered positive if the patient experiences relief of symptoms with active cervical extension^35^. Kase.

Pressure-pain thresholds (PPT) at certain points in the lower back and along with lower limbs were determined by algometry^36^. The examination was carried out twice in the same places, on the left and right sides following the protocol described by Sipko et al.^36^.

### Randomization

During the randomisation process, concealed allocation (ratio 1:1) was performed using 58 printed cards placed in opaque envelopes. Patients were allocated to the treatment with KT or TENS group according to randomised codes. The therapist who prepared the randomisation code by using computer software (XX-X) was not involved in the rest of the study. The therapists who examined the patients (XX-X) for eligibility criteria and collected all baseline demographic and self-report variables and collected all outcome measures during the trial, were blinded to treatment assignment. Due to the nature of the study, patients and physical therapists could not be blinded to treatment assignment. All treatment interventions were carried out by 2 KT instructors with wide clinical experience who were blinded to the outcome measures and baseline examination findings but not to the treatment allocation, although they did not reveal group membership to the physical therapists who gathered outcome measures.

Outcome measures were assessed before the first treatment session (baseline data), and immediately after the 4-week (1 month) intervention period by an assessor blinded to the treatment allocation of the patients.

### Intervention

#### Kinesio taping group (KT)

Patients in this group received the application of KT for the inhibition of lumbar paravertebral plus back exercise program. The tape used in this study was waterproof, porous and adhesive, with a width of 5 cm and a thickness of 0.5 mm.

**Figure 1:**
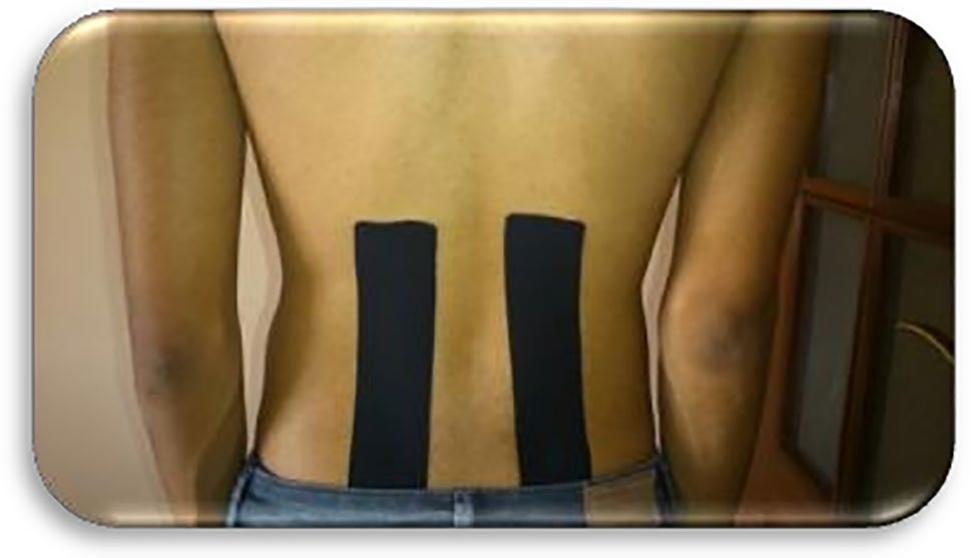
Paravertebral inhibition technique with kinesio taping.

**Figure 2:**
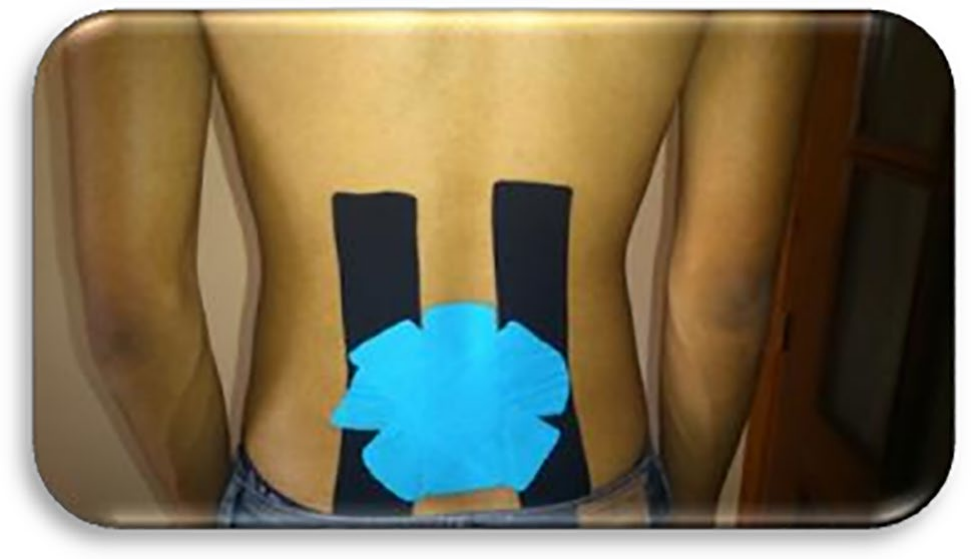
Space correction technique with kinesio taping.

First, two black tape bands, shaped as a capital ‘I’, were placed along the paravertebral musculature, (in a cranial direction), with a length equal to that of the paravertebral musculature of the back, being necessary for the placement of the rest of the band for the patient to perform a little trunk flexion, letting the forearms rest on the treatment table, while the tension-free bands were placed “paper off”. Strips were applied by pressing and adhering the central parts before the ends (Fig. 1). Next, a space correction technique was carried out. With the patient standing, with trunk flexion and support of the forearms on the treatment table, four blue I-strips overlapping in a star shape at 25% tension were placed over the point of maximum pain in the lower back. The middle part of each strip was fixed, and finally, the patient straightened his trunk and from an upright position the anchors were stuck without tension (Fig. 2).

Participants were advised to leave the tape in situ until the next session. Bandages were replaced three times a week during the 4 weeks of intervention.

#### Transcutaneous electrical nerve stimulation (TENS) group

Patients in this group received the application of TENS plus back exercise program. The TENS were administered with a frequency of 30–50 Hz, and phase duration of 200 microseconds, in continuous mode. Four adhesive electrodes were used, which were placed in the lumbar area at a distance of 2 cm from the spinous, forming a square. The current was applied for 40 min with an intensity depending on the tolerance of each patient. The treatment lasted three weekly sessions for 4 weeks.

#### Back exercise program

In both groups, both the application of TENS and the KT bandage were carried out simultaneously a back exercise program by the patients according to the protocol described in a previous study^37^.

The exercise program consisted in eight different exercises according to three kinds of practices to guarantee the stability and control of motor pelvic and, trunk muscle strengthening and stretching. The sequency of the exercises performed is described below:

- Diaphragmatic breathing technique (for 2–3 min).
- Activation of the transverse abdominal muscle (3 × 15 repetitions).
- Pelvic girdle (3 × 15 repetitions).
- Glute bridge (3 × 15 repetitions).
- Erector spinae strengthening (Prone Superman) (3 × 8 − 10 repetitions).
- Front plank (3 × 30 s).
- Side plank (3 × 30 s for each side).
- Lateral leg-raise for gluteus medius (3 × 10 − 15 repetitions).
- Spinal column mobility (Quadruped Cat Camel exercise) (3 × 10 repetitions).

The program was adapted according to the tolerance of the patients. The exercise program was conducted by a physical therapist. The patients performed 10–15 repetitions of each exercise in the set, once a day, three times a week for a total of 4 weeks.

#### Sample size

We used the Ene 3.0 software (Autonomous University of Barcelona, Spain) to calculate the sample size. We based the calculations on the detection differences of 2.5 points in the RMQ (minimal clinically important difference)^38^, assuming a standard deviation (SD) of 2.5 points, a two-tailed test, an alpha (α) level of 0.05, and a desired power (beta) of 80%. The estimated desired sample size was calculated in 28 subjects per group.

### Statistical analysis

Statistical analysis was performed using SPSS statistical software, version 20.0, conducted according to the intention-to-treat analysis principle.

Key baseline demographic variables and self-report measures were compared between groups using independent *t-*tests for continuous data and chi-square tests for categorical data. To test for homogeneity of variances Levene test was performed, with a 95% confidence interval.

Separate 2 × 2 mixed model ANCOVA with repeated measurements need to be conducted in order to test the effect of the treatment on disability as primary outcome and fear symptoms, anxiety, depression, sleep quality and pain as secondary outcomes with time (baseline and 4-week follow-up) as within-subject variable and group (KT or TENS) as between-subjects variable.

Changes in variable scores within and between groups were measured by means (95% confidential interval) of t-tests for paired or independent samples as appropriate. The effect size was calculated according to Cohen’s d statistic. An effect size < 0.2 reflects a negligible difference, between ≥ 0.2 and ≤ 0.5 a small difference, between ≥ 0.5 and ≤ 0.8 a moderate difference, and ≥ 0.8 a large difference. *P* < 0.05 was considered significant in all tests.

### Ethics approval and consent to participate

The study was approved by the bioethics committee of the University of Granada (Spain) on 2016 and complied with the 2013 modification of the Helsinki Declaration and Spanish legislation for clinical trials (ClinicalTrials.gov Identifier: NCT02812459).

### Consent for publication

Informed consent was obtained from all the participants before their involvement.

## Results

**Figure 3:**
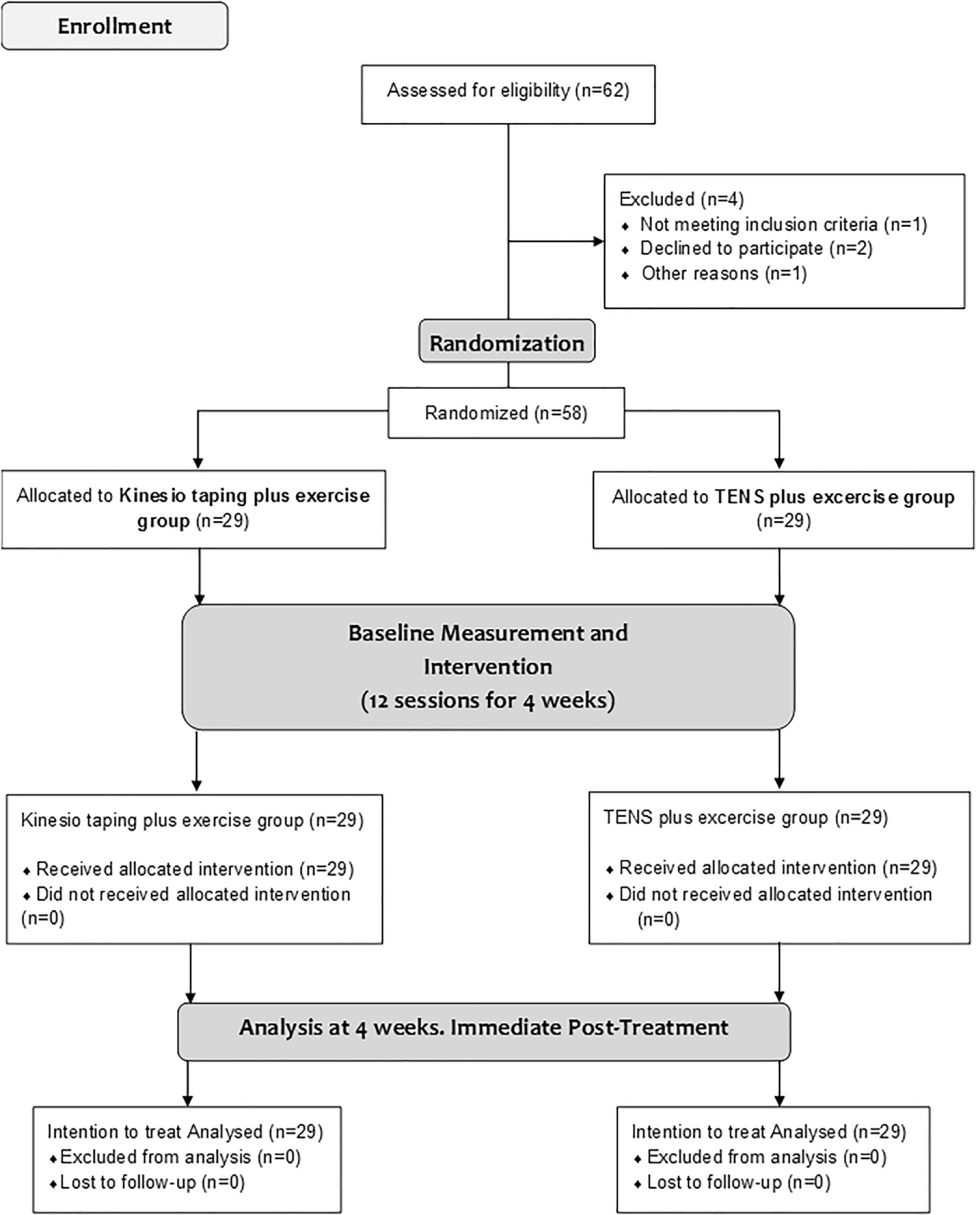
Flow diagram of the recruitment and follow-up of patients throughout the study.

Of the 62 patients recruited for the study, 58 patients with an average age of 43.41 years (SD = 17.62) met the inclusion criteria and were randomly assigned to the experimental (n = 29) or control group (n = 29). A CONSORT flow diagram of the participants throughout the study is shown in Fig. 3.

The baseline characteristics of participants in each group are shown in Table 1. There were no significant differences between the groups for these variables (*P* ≥ 0.101).

**Table 1:** Characteristics of patients at baseline.

	Kinesio Taping Group n = 29	TENS Group n = 29	*P*-value		
Age (y) (mean ± SD)	44 ± 9	46 ± 5	0.120		
Cigarettes/week (mean ± SD)	11.22 ± 11.87	13.55 ± 14.68	0.518		
Cups of coffee/week (mean ± SD)	6.18 ± 5.56	8.90 ± 6.51	0.101		

	Freq	%	Freq	%	
Women	17	58.62	21	72.41	0.570
Acute discomfort in the last 2 years	7	24.13	11	37.93	0.399
Difficulty sleeping	11	37.93	13	44.82	0.793
Sleep disturbances due to pain	8	27.59	10	34.48	0.779

Values are expressed as absolute and relative frequencies for categorical variables and as means ± standard deviation for continuous variables.

Cigarettes/week (cigarettes smoked per week), cups of coffee/week (cups of coffee drunk per week).

### Self-reported outcomes

The Group * Time interaction for the 2 × 2 mixed ANCOVA did not show significantly difference between the score of both groups in disability due to CLBP (*F* = 0.21; *P* < 0.884), kinesiophobia (*F* = 2.094; *P* = 0.154) or the level of functional impediment patients experienced as a result of back pain (*F* = 1.001; *P* = 0.321). However differences between groups for the NPRS (*F* = 4.169; *P* = 0.046) were found, where the TENS group showed a greater decreased.

A large effect size was observed in TENS group for disability, pain and kinesiophobia (d ⩾1.09). Similar results were found in kinesio taping group (d ⩾1.65) with the exception of kinesiophobia that showed a moderate difference. Table 2.

**Table 2:** Baseline, post-treatment, pre-post-treatment differences and change scores in each group (95% confidence interval) for self-reported outcomes: disability, kinesiophobia and pain.

Outcome/group	Baseline	One month post-treatment	Cohen’s *d*	Within-group score change	Between-group score change
**RMDQ (− 24)**					
Kinesio	8.07 ± 4.26	5.85 ± 4.93	1.34	2.22 (1.20, 3.24)	− 1.06 (− 3.55, 1.43)
TENS	7.14 ± 4.93	4.79 ± 4.32	1.76	2.35 (0.99, 3.70)	
**ODI (0–5)**					
Kinesio	19.11 ± 15.31	11.52 ± 12.38	2.05	7.59 (4.60, 10.59)	− 0.83 (− 7.92, 6.26)
TENS	21.04 ± 17.73	10.69 ± 14.06	1.71	10.34 (5.67, 15.02)	
**NPRS (0–10)**					
Kinesioc Kn	6.19 ± 2.29	4.07 ± 3.06	1.65	2.11 (1.08, 3.14)	− 1.83 (− 3.28, − 0.39)*
TENS	5.79 ± 2.96	2.24 ± 2.31	2.71	3.55 (2.54, 4.57)	
**TSK (17–68)**					
Kinesio	25.70 ± 6.34	24.37 ± 7.14	0.67	1.33 (− 0.26, 2.93)	− 0.75 (− 4.86,3.36)
TENS	27.04 ± 6.46	23.62 ± 8.19	1.09	3.41 (0.99, 5.84)	

Values are expressed as means ± standard deviation for baseline and 1 month post-treatment and as mean (95% confidence interval) for within- and between-group change scores/*Significant Group * Time interaction (ANCOVA, *p* < 0.05).

*RMDQ* the Roland Morris disability questionnaire), *ODI* Oswestry disability index, *NPRS* change from baseline numerical pain rating scale, *TSK* Tampa scale for kinesiophobia.

Pair-wise comparisons with baseline values demonstrated significant differences in pain (*P* < 0.001), disability (*P* = 0.001) and kinesiophobia (*P* = 0.007) variables at 4 weeks post-treatment in the TENS group (*P* = 0.001). In the KT group only disability (*P* < 0.001) and pain (*P* < 0.001), outcomes were significant. The score change in pain was significantly greater in the TENS group (NPRS 3.55) than in the KT group (NPRS 2.11) (Table 2).

### Physical outcomes

At the end of the 4-week treatment period, the Group * Time interaction for the 2 × 2 mixed ANCOVA for mechanosensitivity (SLR y ST), did not show significantly difference for right SLR (*F* = 0.065; *P* = 0.801), left SLR (*F* = 0.076; *P* = 0.785), right ST (*F* = 1.280; *P* = 0.263), left ST (*F* = 0.024; *P* = 0.877) scores. Also did not show significantly differences for PPT scores, in the spinal, left spinal, right gluteus medius, left gluteus medius, right sural triceps, left sural triceps, right anterior tibialis and left anterior tibialis (*F* ≤ 2.002; *P* ≤ 0.809).

The treatment showed a moderate-large effect for right ST and a large effect for left ST, and all the PPT in the KT group, but did not show any effect on SLR (right or left). The TENS group showed, a moderate-large effect for right and left SLR and ST and a large effect for all the PPT. Table 3.

**Table 3:** Baseline, post-treatment, pre-post-treatment differences and change scores in each group (95% confidence interval) for physical outcomes.

Outcome/group	Baseline	One month post-treatment	Cohen’s *d*	Within-group score changes	Between-group score changes
**SLR (s)**					
Kinesio					
Right	60.87 ± 24.09	63.47 ± 17.44	0.20	− 2.600 (− 17.62,12.42)	0.12 (− 13.38,13.62)
Left	63.60 ± 19.96	66.60 ± 14.07	0.24	− 3.00 (− 17.55, − 11.55)	− 1.54 (− 13.29,10.22) 12.69)*
**TENS**					
Right	58.94 ± 20.66	63.59 ± 19.96	0.54	− 4.65 (− 13.85,4.56)	
Left	61.40 ± 15.74	66.73 ± 17.05	0.56	− 5.33 (− 16.27, 5.60)	
**ST**					
Kinesio					
Right	169.19 ± 34.24	174.93 ± 23.55	0.50	− 5.74 (− 14.98,3.50)	− 4.24 (− 20.13,11.66)
Left	161.63 ± 44.54	179.82 ± 0.96	0.83	− 18.19 (− 35.84, − .53)	− 8.95 (− 21.96,4.05)
TENS					
Right	154.69 ± 51.14	170.69 ± 34.92	0.79	− 16.00 (− 31.74, − .26)	
Left	150.52 ± 54.38	170.86 ± 34.17	0.71	− 20.34 (− 42.49,1.80)	
**PPT L5-S1**					
Kinesio					
Right	4.28 ± 2.99	5.33 ± 3.33	0.82	− 1.05 (− 2.10, − 0.12)	− 0.10 (− 1.74, 1.53)
Left	4.49 ± 3.35	5.70 ± 3.61	1.29	− 1.21 (− 1.96,0.45)	− 0.34 (− 2.09,1.43)
TENS					
Right	3.35 ± 2.21	5.23 ± 2.70	2.33	− 1.88 (− 2.50, − 1.25)	
Left	3.93 ± 2.14	5.36 ± 2.86	1.40	− 1.44 (− 2.23, − .64)	
**PPT gluteus medius**					
Kinesio					
Right	4.84 ± 3.56	6.49 ± 3.46	1.49	− 1.65 (− 2.55, − .76)	− 0.55 (− 2.37,1.26)
Left	4.69 ± 3.35	6.23 ± 3.67	1.37	− 1.54 (− 2.44,−.63)	− 0.54 (− 2.37, 1.29)
TENS					
Right	3.98 ± 2.65	5.94 ± 3.29	1.57	− 1.96 (− 2.92, − .99)	
Left	4.01 ± 2.45	5.69 ± 3.11	1.59	− 1.68 (− 2.50, − .86)	
**PPT sural triceps**					
Kinesio					
Right	4.30 ± 3.00	5.40 ± 2.97	2.10	− 1.10 (− 1.53, − .68)	− 0.45 (− 1.97, 1.07)
Left	4.14 ± 2.62	4.93 ± 2.51	1.32	− 0.79 (− 1.27, − .31)	0.14 (− 1.26, 1.54)
TENS					
Right	3.59 ± 2.18	4.96 ± 2.68	1.94	− 1.36 (− 1.91, − .82)	
Left	3.92 ± 2.67	5.07 ± 2.72	1.03	− 1.14 (− 2.00, − .28)	
**PPT anterior tibialis**					
Kinesio					
Right	4.97 ± 3.17	5.82 ± 3.02	0.83	− .85 (− 1.68, − .26)	− 0.41 (− 1.96, 1.14)
Left	4.96 ± 3.17	6.03 ± 3.03	1.45	− 1.07 (− 1.66, − .48)	− 0.64 (− 2.29, 1.01)
TENS					
Right	4.16 ± 2.46	5.41 ± 2.72	1.88	− 1.26(− 1.77, − .74)	
Left	4.03 ± 2.08	5.40 ± 3.14	2.24	− 1.37 (− 1.84, − .90)	

Values are expressed as means ± standard deviation for baseline and 1 month post-treatment and as mean (95% confidence interval) for within- and between-group change scores/*Significant Group * Time interaction (ANCOVA, *p* < 0.05).

*SLR* straight leg raise, *ST* slump tests, *PPT L5-S1* pressure-pain thresholds at the level of L2, 3 cm away from the interspinous line, *PPT* gluteus medius (musculus gluteus medius), *PPT* sural triceps (musculus triceps surae), *PPT* anterior tibialis (musculus tibialis anterior).

Pair-wise comparisons with baseline values demonstrated significant differences at 4 weeks post-treatment in the KT in disability (*P* < 0.001), left ST (*P* = 0.044), PPT scores of right L5-S1 (*P* = 0.048), left L5-S1 (*P* = 0.003), right gluteus (*P* = 0.001), left gluteus (*P* = 0.002), right sural triceps (*P* < 0.001), left sural triceps (*P* = 0.021), right anterior tibial (*P* = 0.044) and left anterior tibial (*P* = 0.001). In the TENS group the results showed significant differences in right ST (*P* = 0.047), PPT scores in the right L5-S1 (*P* < 0.001), left L5-S1 (*P* = 0.001), gluteus (*P* < 0.001), right sural triceps (*P* < 0.001), left sural triceps (*P* = 0.011) and anterior tibial (*P* < 0.001). The improvements in these scores before and after treatment were not significantly different between groups. (Table 3).

### Mental health and sleep quality

The Group * Time interaction for the 2 × 2 mixed ANCOVA did not show significantly difference between the score of both groups in anxiety (*F* = 0.286; *P* = 0.595) or depression (*F* = 1.107; *P* = 0.298). For the PSQI there were no significantly differences between total score, subjective sleep quality, sleep latency, sleep duration, sleep efficiency, sleep disturbance, sleeping medication or daytime dysfunction (*F* ≤ 2.213; *P* ≤ 0.962) scores.

Both groups showed a large effect on anxiety and depression, however its effect on some sleep quality variables was small-moderate, duration and efficiency for the KT group and efficiency and the use of mediation for the TENS group. The rest of the sleep quality variables showed a large effect for both groups. (Table 4).

**Table 4:** Baseline, post-treatment, pre-post-treatment differences and change scores in each group (95% confidence interval) for mental health and sleep quality.

Outcome/group	Baseline	One month post-treatment	Cohen’s *d*	Within-group score change	Between-group score change
**Anxiety**					
Kinesio	12.59 ± 11.92	8.00 ± 11.95	1.74	4.59 (2.47, 6.72)	0.48 (− 5.02, 5.99)
TENS	12.28 ± 8.57	8.48 ± 7..96	1.34	3.79 (1.59, 5.99)	
**Depression**					
Kinesio	9.70 ± 9.37	5.93 ± 8.01	1.65	− 3.78 (1.93, 5.63)	− 0.64 (− 3.74, 3.61)
TENS	8.48 ± 5.22	5.86 ± 5.23	1.52	2.62 (1.28, 3.96)	
**Sleep Quality**					
Kinesio					
Total	8.30 ± 4.1	6.26 ± 3.64	1.63	2.04 (1.03, 3.05)	− 1.19 (− 3.05, 0.67)
Subj	1.33 ± 0.68	1.00 ± 0.69	1.10	0.33 (0.09,0.58)	− 0.35(− 0.72, 0.03)
Laten	1.59 ± 0.75	1.29 ± 0.87	0.99	0.30 (0.06, 0.54)	0.30 (− 0.76, 0.16)
Durat	1.22 ± 1.01	1.11 ± 0.93	0.39	0.11 (− .12,0.34)	− 0.22 (− 0.69, 0.26)
Effici	0.30 ± 0.67	0.41 ± 0.69	0.32	− .11 (− .39, 0.17)	− 0.27 (− 0.57, 0.03)
Distu	1.56 ± 0.58	1.22 ± 0.58	1.41	− .33 (0.14, 0.52)	0.16 (− 0.19, 0.51)
Medi	1.00 ± 1.21	0.48 ± 0.89	1.08	0.52 (0.13, 0.90)	− 0.21 (− 0.65, 0.24)
Dysfu	1.30 ± 0.91	0.82 ± 0.79	1.22	0.48 (0.16, 0.80)	− 0.26 (− 0.71, 0.18)
**TENS**					
Total	7.14 ± 3.31	5.07 ± 3.27	1.74	2.07 (1.15, 2.99)	
Subj	1.17 ± 0.81	0.66 ± 0.72	1.66	0.52 (0.28, 0.76)	
Laten	1.35 ± 0.86	1.00 ± 0.85	0.82	0.34 (0.2, 0.67)	
Durat	1.21 ± 0.90	0.90 ± 0.82	1.05	0.31 (0.81, 0.54)	
Effici	0.21 ± 0.62	0.14 ± 0.35	0.31	0.69 (− 0.11, 0.24)	
Distu	1.72 ± 0.75	1.34 ± 0.73	0.91	0.35 (0.05, 0.64)	
Medi	0.45 ± 0.99	0.28 ± 0.75	0.46	0.17 (− 0.12, 0.46)	
Dysfu	1.04 ± 0.73	0.66 ± 0.67	1.06	0.38 (0.10, 0.66)	

Values are expressed as means ± standard deviation for baseline and 1 month post− treatment and as mean (95% confidence interval) for within- and between-group change scores.

*Anxiety* beck’s anxiety inventory, *Depression* beck’s depression inventory, *Sleep Quality* the pittsburgh sleep quality index, *Subj* subjective sleep quality, *Laten* sleep latency, *Durat* sleep duration, *Effici* habitual sleep efficiency, *Distu* sleep disturbances, *Medi* use of sleep medications, *Dysfu* diurnal dysfunction.

Pair-wise comparisons with baseline values demonstrated significant differences in anxiety (*P* = 0.001), depression (*P* < 0.001), sleep quality (*P* < 0.001), subjective sleep quality (*P* < 0.001), sleep latency (*P* = 0.039), sleep duration (*P* = 0.010), sleep perturbation (*P* = 0.023), and dysfunction (*P* = 0.009) variables at 4 weeks post-treatment in the TENS group and for anxiety (*P* < 0.001), depression (*P* < 0.001), sleep quality (*P* < 0.001), subjective sleep quality (*P* = 0.010), sleep latency (*P* = 0.018), sleep perturbation (*P* = 0.001), medication (*P* = 0.010), and dysfunction (*P* = 0.004) scores in KT group. The improvements in these scores before and after treatment were not significantly different between groups (Table 4).

## Discussion

One month of treatment through a back exercise program combined with KT or TENS on patients with CLBP showed an improvement in disability, intensity of pain, anxiety, depression, sleep pattern and pain thresholds. However, the combination of exercises with TENS improved more significantly the intensity of pain, disability and kinesiophobia at the end of the 4-week course of treatment.

CLBP is a significant health problem with high prevalence worldwide. It is associated with huge costs for society^39^. Clinical practice guidelines show many of the interventions available to treat patients with CLBP, but the vast majority of interventions have a modest effect in reducing pain and disability^40^.

An intervention that has been widespread in recent years is the use KT^17,41^. KT is not recommended to be used as an isolated intervention in people with CLBP^42^. As recent studies that investigated the effect of a combination of exercise and KT on pain and stability in patients with CLBP^17,42,43^, our findings in terms of LBP pain were consistent with these results, which observing a highly significant difference in pain reduction, after 4 weeks of treatment with KT in conjunction with exercise. Two recent systematic reviews found that the exercise treatments used were heterogeneous and varied in terms of the type of exercise, the program designed, the dose (duration, frequency, intensity), the administration format (e.g. clinician supervised, group), and whether they were combined with other conservative treatments, therefore, the comparison of our results with other previous studies should be taken with caution^44,45^.

Although the mechanism through which KT acts on the conditions of the musculoskeletal system is still unclear, the most accepted hypothesis is that KT applies pressure to the skin or stretches the skin and that this external load can stimulate cutaneous mechanoreceptors (large fibers myelinated) and therefore inhibit pain transmission according to the theory of door control^23,46^.

In recent years, it has been theorized that this type of bandage can be useful to achieve an analgesic effect on the spine. A recent systematic review analysed this effect in patients with CLBP, finding statistically significant differences regarding the degree of pain between the group to which KT was applied and the group to which a placebo was applied^17^. In fact, a decrease in pain has been reported after the specific use of KT application (origin to insertion) in different pathologies^47–50^. However, regarding the methodological quality of the selected articles, we found serious limitations in terms of the fulfilment of the defined criteria.

Concerning another of the techniques used in our study, some studies show that TENS therapy was effective in pain relief^49^. TENS is a common modality for the treatment of musculoskeletal pain^51^. According to the door control theory^23^, TENS can stimulate large diameter afferent fibers, which can reduce the transmission of pain signals through the small nociceptive of afferent fibers, thus inhibiting pain discrimination and perception. In our study, it has been observed how the TENS have produced a significant improvement, in the threshold of pressure pain (in the intragroup analysis, based on pre-post-treatment), in all points examined bilaterally (L5-S1, gluteus medius, anterior sural and tibial triceps).

People with LBP usually show a 6-point improvement in the ODI^27^. Our estimation of the effect of KT on disability measures on the ODI is 7.5 points, which is a relatively good score compared to the gamma of possible scores on the ODI^27^ and compared to the initial score of the study participants.

For the TSK questionnaire, a variation of 1.33 points has been observed in patients treated with KT. In the article by Castro-Sánchez et al.^52^, the measurement of this variable is also reflected, showing a change of 2 points at 4 weeks after applying the treatment.

In both therapies, the presence of short-term placebo analgesic effects in response to the simulated control should support the use of the placebo protocol. The placebo analgesic responses are modulated through expectations regarding the treatment of pain and are regulated through responses to harmful stimuli in the spinal cord and brain, as well as the activation of descending pain by inhibitory pathways^53^.

The present study has some limitations. Firstly the small sample of patients, which may not be representative of the entire population of individuals with nonspecific CLBP thus affecting external validity. Secondly, we only investigated the short-term results of analgesic currents and a certain type of bandage with KT, and we could not conclude their longer-term effects, which deserve future research through randomized clinical trials. Finally, including a third group that only involves the performance of therapeutic exercise could help to better understand the individual contributions made by electroanalgesia and bandage to said therapy.

In conclusion, individuals with non-specific CLBP experienced a significant improvement in pain intensity and disability after receiving 12 treatment sessions that combined a back exercises program with KT or TENS, being greater in the group treated with TENS application. More future researches are needed to evaluate the effects of KT and electrotherapy over a longer period to observe the long-term effects.

## Data Availability

The datasets used and/or analysed during the current study are available from the corresponding author on reasonable request.
